# Drug-Targeted Inhibition of Peroxisome Proliferator-Activated Receptorγ Enhances the Chemopreventive Effect of Anti-Estrogen

**DOI:** 10.18632/oncotarget.457

**Published:** 2012-04-13

**Authors:** Hongyan Yuan, Levy Kopelovich, Yuzhi Yin, Jin Lu, Robert I. Glazer

**Affiliations:** ^1^ Department of Oncology, Georgetown University School of Medicine, and Lombardi Comprehensive Cancer Center, Washington, DC; ^2^ Chemoprevention Branch, National Cancer Institute, Bethesda, MD; ^3^ Laboratory of Allergic Diseases, National Institute of Allergy and Infectious Diseases, Bethesda, MD

**Keywords:** PPARγ, ERα, fulvestrant, GW9662

## Abstract

The peroxisome proliferator-activated receptorγ (PPARγ) is a key regulator of metabolism, proliferation, inflammation and differentiation, and upregulates tumor suppressor genes, such as PTEN, BRCA1 and PPARγ itself. Examination of mammary carcinogenesis in transgenic mice expressing the dominant-negative Pax8PPARγ fusion protein revealed that tumors were estrogen receptorα (ER)-positive and sensitive to the ER antagonist, fulvestrant. Here we evaluated whether administration of an irreversible PPARγ inhibitor in vivo could similarly induce ER expression in otherwise ER-negative mammary tumors following induction of carcinogenesis, and sensitize them to the antitumor effects of fulvestrant. In addition, we wished to determine whether the effect of GW9662 was associated with a PPAR-selective gene expression profile. Mammary carcinogenesis was induced in wild-type FVB mice by treatment with medroxyprogesterone and dimethylbenz(a)anthracene (DMBA) that were subsequently maintained on a diet supplemented with 0.1% GW9662, and tumorigenesis and gene expression profiling of the resulting tumors were determined. Administration of GW9962 resulted in ER+ tumors that were highly sensitive to fulvestrant. Tumors from GW9662-treated animals exhibited reduced expression of a metabolic gene profile indicative of PPARγ inhibition, including PPARγ itself. Additionally, GW9662 upregulated the expression of several genes associated with the transcription, processing, splicing and translation of RNA. This study is the first to show that an irreversible PPARγ inhibitor can mimic a dominant-negative PPARγ transgene to elicit the development of ER-responsive tumors. These findings suggest that it may be possible to pharmacologically influence the responsiveness of tumors to anti-estrogen therapy.

## INTRODUCTION

The peroxisome proliferator-activated receptor (PPAR) nuclear receptor subfamily regulates a number of metabolic processes, including fatty acid β-oxidation, glucose utilization, cholesterol transport, energy balance and adipocyte differentiation [1-4]. PPARs also play important roles in modulating inflammation, proliferation, angiogenesis and neoplasia [5-8]. PPARs function as heterodimeric partners with RXR, and require high-affinity binding of PPAR isotype-specific ligands to engage transcription. Of the three subtypes, PPARγ is the major species expressed in the mammary gland and in primary and metastatic breast cancer and breast cancer cell lines [5].

PPARγ and PPARδ modulate cell fate in the mammary gland [6, 9, 10], suggesting that PPAR agonists or antagonists may have the potential to regulate differentiation and hence tumor progression. PPARγ agonists are potent chemopreventive agents in mammary carcinogenesis [11], which is consistent with the enhancement of mammary tumorigenesis by PPARγ heterozygosity [12]. In a large percentage of follicular thyroid cancers, PPARγ exists as the dominant-negative fusion protein, Pax8-PPARγ, associated with the t(2;3)(q13;p25) translocation [13]. Pax8PPARγ potently blocks PPARγ function [13, 14], rather than merely serving as a low affinity receptor that can be activated at high ligand concentrations [15]. Importantly, the irreversible PPARγ ‘suicide’ inhibitor, GW9662 [16], mimics the growth promoting effects of Pax8PPARγ in thyroid cells [17], suggesting that selective pharmacological manipulation of PPARγ is feasible.

Although many studies have addressed the interactions between different nuclear receptor subfamilies, an area of relevance to breast cancer is the inhibitory effect of PPARγ on ERα (ER) promoter activation through its interaction with ER response elements [18]. Conversely, ER may bind to PPARγ response elements (PPREs) to inhibit PPAR-dependent transcription [19]. The ER and PPARγ pathways produce opposite effects on PI3K/AKT signaling, accounting in part, for the divergent responses produced by their cognate ligands in estrogen-dependent human breast cancer cells [19]. These findings suggest that suppression of PPARγ may upregulate ER expression in tumors to allow the implementation of anti-estrogen therapy. As a proof of principle, this was demonstrated by the effectiveness of the ER antagonist, fulvestrant, in preventing mammary tumorigenesis in MMTV-Pax8PPARγ mice, in which tumors normally present with a more aggressive progenitor cell phenotype [10]. Therefore, from a chemoprevention perspective, it would be important to be able to mimic the MMTV-Pax8PPARγ transgene pharmacologically by administering a PPARγ antagonist to increase the percentage of ER^+^ tumors and render them amenable to anti-estrogen therapy. This approach would be dependent on whether a PPAR antagonist could be developed with favorable specificity and pharmacokinetic properties to achieve selective and sustained inhibition of PPARγ. Examples of PPARγ antagonists are the suicide inhibitors, GW9662 (2-chloro-5-nitro-*N*-phenylbenzamide) [16], 2-bromo-5-nitro-*N*-phenylbenzamide [20] and the structurally similar T0070907 [21], as well as the partial PPARγ agonists, GW0072 [22] and L-764406 [23]. Although, GW9662 and T0070907 have also been reported to produce off-target effects *in vitro* [24-26], their *in vivo* selectivity has yet to be demonstrated. In this report, we show that GW9662 when administered continuously in the diet beginning at the onset of mammary carcinogenesis induces ER-responsive tumors susceptible to fulvestrant therapy. Furthermore, GW9662 inhibited a PPARγ-dependent metabolic gene expression signature, including PPARγ itself. These results are the first to demonstrate that GW9662 is at least in part PPARγ-selective, and can induce sensitivity to anti-estrogen therapy.

## RESULTS

### GW9662 induces sensitivity to antiestrogen therapy

**Figure 1:**
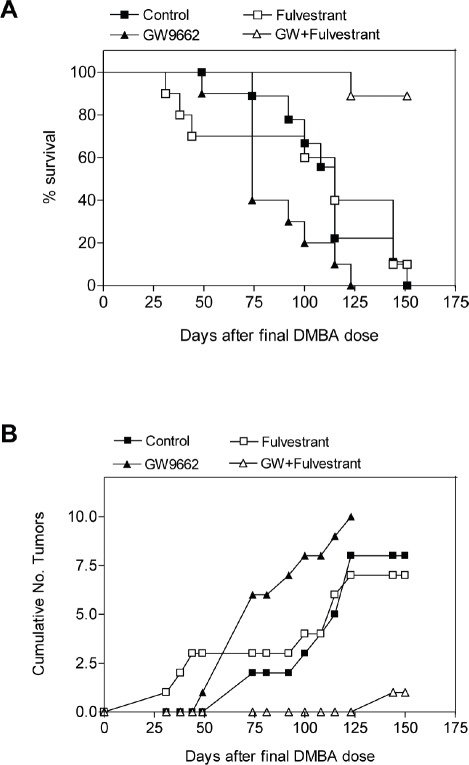
GW9662 enhances the sensitivity of mammary tumors to fulvestrant (A) Survival curves of mice administered a control diet, a diet supplemented with 0.1% (w/w) GW9662, 250 mg/kg fulvestrant administered s.c. every other week or the combination of the GW9662 diet and fulvestrant. GW9662 treatment alone produced a significant reduction in survival vs. control mice (*P*=0.0382), but not vs. fulvestrant treatment (*P*=0.0759); fulvestrant treatment alone did not significantly affect survival (*P*=0.7223). GW9662 and fulvestrant treatment produced a significant increase in survival vs. fulvestrant (*P*=0.0008) or GW9662 (*P*=0.0001) treatment alone. Each group contained 10 mice. Statistical significance was determined by the log rank test. (B) Tumor formation in the experimental groups indicated in (A). Neither GW9662 (*P*=0.3942) nor fulvestrant (*P*=0.3339) treatment alone significantly affected tumor number vs. control mice. GW9662 and fulvestrant treatment produced a significant reduction in tumor number vs. either fulvestrant (*P*=0.0001) or GW9662 (*P*=0.0004) treatment alone. Each group contained 10 mice. Statistical significance was determined by the two-tailed Student's t test.

**Figure 2:**
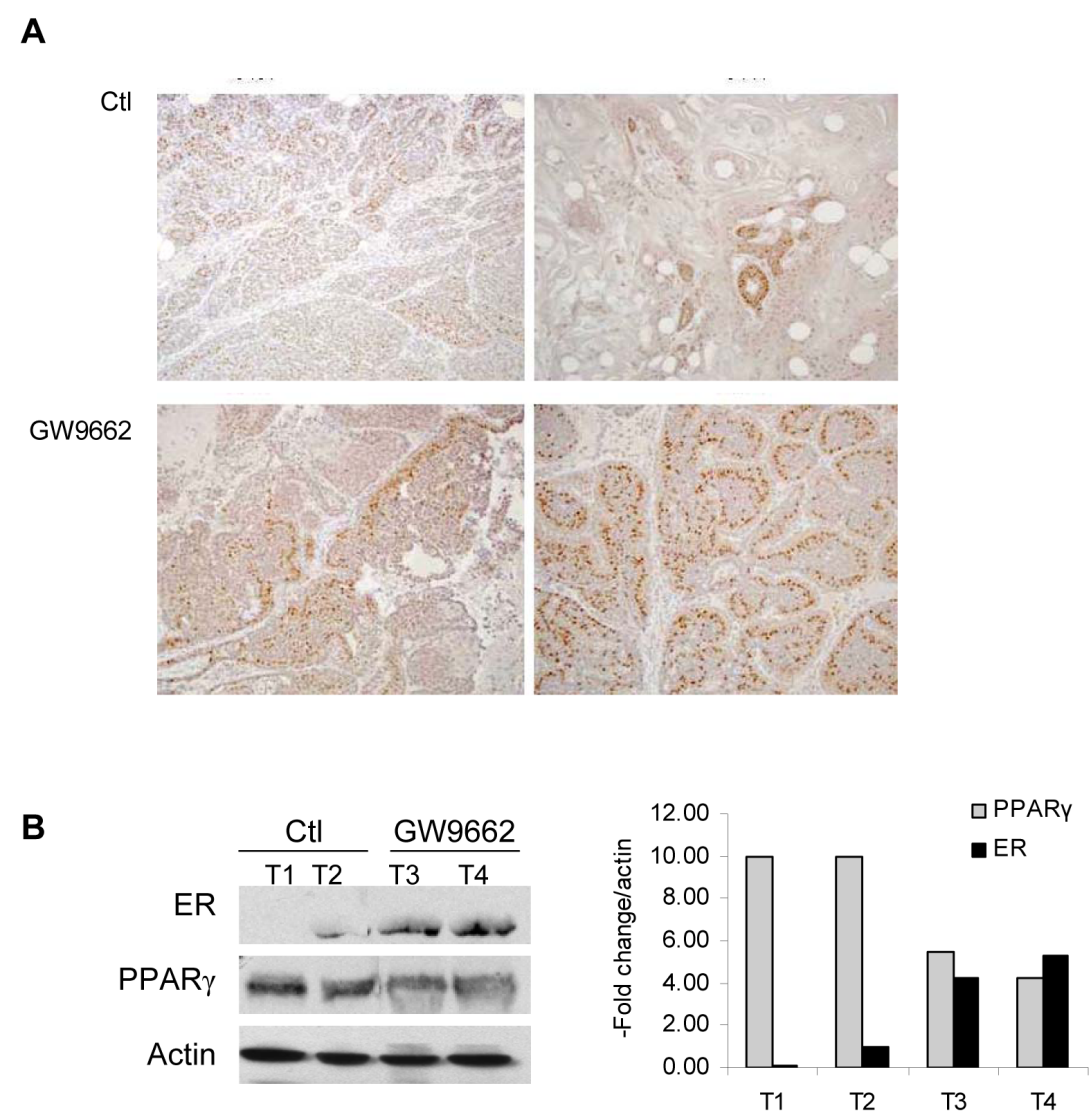
ER expression in adenocarcinomas from control and GW9662 mice (A) Immunohistochemical detection of ER expression. Two representative tumors from control and GW9662-treated mice are shown. ER expression was increased following GW9662 treatment. Magnification 200X. (B) Western analysis of ER and PPARγ expression. Two representative tumors from control and GW9662-treated mice are shown. ER expression was increased, and PPARγ expression reduced following GW9662 treatment. The bar graph represents quantitation of the western blot normalized to actin expression.

**Figure 3:**
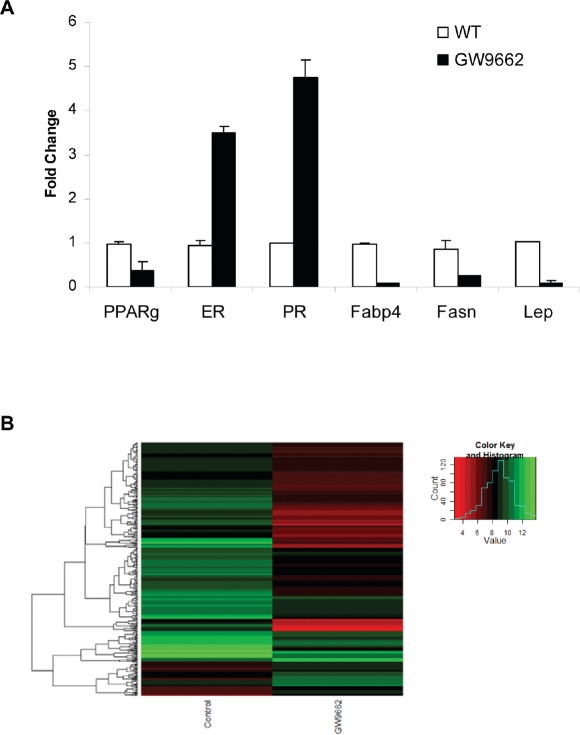
(A) qRT-PCR analysis of gene expression in adenocarcinomas from control and GW9662-treated mice Gene selection was based on the data in Table 1. (B) Heat map of changes in gene expression based on the data in Table S2.

To evaluate the chemopreventive effect of GW9662 on mammary tumor development, carcinogenesis was induced in FVB mice by progestin and DMBA treatment. Animals were maintained on either a control diet or a diet supplemented with 0.1% GW9662 beginning one day after the last dose of DMBA, and both groups were administered either vehicle or 250 mg/kg fulvestrant by subcutaneous injection every other week (Figure 1). Animals maintained on GW9662 alone exhibited a modest reduction in survival (Figure 1A) similar to what was observed previously in MMTV-Pax8PPARγ transgenic mice [10], but not a reduction in the total number of tumors (Figure 1B). While no significant difference in survival was noted for fulvestrant-treated control mice, a marked increase in survival (Figure 1A) and a reduction in tumor number (Figure 1B) were observed in animals maintained on GW9662 and treated with fulvestrant. Consistent with these findings was an increase in ER expression in tumors from GW9662-treated mice in comparison to animals maintained on the control diet as determined by immunohistochemical (Figure 2A) and western analyses (Figure 2B). Increased ER, as well as PR expression, was accompanied by an increase in Esr1 and Pgr mRNA levels (Figure 3A). GW9662 treatment also resulted in a reduction of PPARγ protein (Figure 2B) and mRNA (Figure 3A). Histological evaluation of the tumors indicated that GW9662, but not fulvestrant, produced a significant increase in the percentage of adenocarcinomas (P=0.0333) (Table S1).

### Gene expression analysis

**Figure 4:**
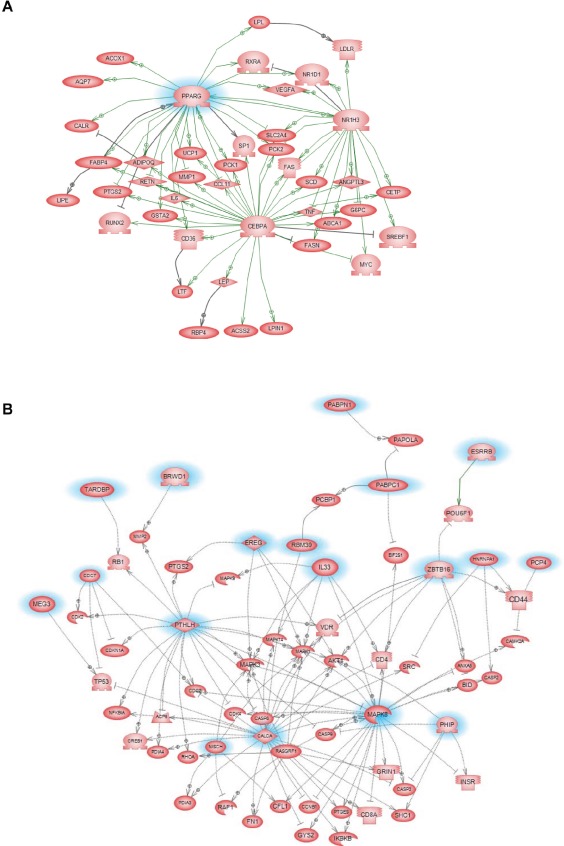
GW9662 signaling pathways in tumors from control and GW9662-treated animals Pathways are based on the expression of genes that were reduced ≥2.5-fold by GW9662 in Table S1. Metabolic signaling pathways associated with genes that were downregulated by GW9662.

Gene microarray analysis of tumors from control and GW9662-treated animals indicated that 356 genes were differentially affected by GW9662 treatment (Figure 3B). Of the 303 genes downregulated by GW9662, 24% were metabolic genes, and 55% of which contain PPREs (Table 1). In addition, there were 10 genes regulated by transcription factors Cebpa and Pouf1, which are PPAR-regulated. Overall, 67% of the metabolic genes were directly or indirectly regulated by GW9662. Gene ontology of the differentially expressed genes (Table S1) indicated that the largest percentage were associated with transport, glucose and lipid metabolism, and developmental processes (Table 2). Pathway linkage analysis revealed that most of the genes whose expression was downregulated by GW9662 were linked directly or indirectly to PPARγ (Figure 4), whereas, those genes whose expression was increased by GW9662 were connected to Mapk3, Mapk8 and Akt signaling (Figure S1). Interestingly, the majority of the genes upregulated by GW9662 were associated with transcription, splicing, processing and translation of RNA (Table S2). In particular, RBM39, whose expression was increased 6.6-fold by GW9662, was recently reported to be increased in ER-dependent mammary tumors developing in caveolin-1 knockout mice [27].

**Table 1:** Metabolic genes downregulated by GW9662 Shown are genes whose signal was >300 in either group and were changed [3] 2.5-fold in GW9662-treated animals vs. control. The full list of changes in gene expression are presented in Table S2. Gene symbols in bold contain PPREs.

			Raw Value	
Gene symbol	Gene Title	Fold Change	WT	GW9662
**Ces3**	carboxylesterase 3	−105.7	2733	25
**Gys2**	glycogen synthase 2	−74.7	558	7
**Lep**	leptin	−74.7	1231	16
**Aqp7**	aquaporin 7	−55.6	3523	63
**Pnpla3**	patatin-like phospholipase domain containing 3	−53.9	1301	24
Cox8b	cytochrome c oxidase, subunit VIIIb	−51.0	1324	26
Cyp2e1	cytochrome P450 family 2, subfamily e, polypeptide 1	−44.3	5209	118
**Pck1**	phosphoenolpyruvate carboxykinase 1, cytosolic	−43.9	3071	70
**Retn**	resistin	−35.7	10637	298
**Rbp4**	retinol binding protein 4, plasma	−33.9	3187	94
Lao1	L-amino acid oxidase 1	−30.2	3092	103
**Fabp3**	fatty acid binding protein 3, muscle and heart	−27.0	886	33
**Cd36**	CD36 antigen	−22.5	6726	306
**Car4**	carbonic anhydrase 4	−22.3	982	44
**Fabp4**	fatty acid binding protein 4, adipocyte	−21.7	7777	3543
**Adipoq**	adiponectin, C1Q and collagen domain containing	−21.6	10299	522
**Adig**	adipogenin	−20.8	1676	85
**Acsl1**	acyl-CoA synthetase long-chain family member 1	−18.6	3172	374
**Lipe**	lipase, hormone sensitive	−16.2	1329	82
**Hsd11b1**	hydroxysteroid-11-beta dehydrogenase 1	−15.7	28.15	1.79
**Pparg**	peroxisome proliferator activated receptor gamma	−13.9	967	69
Pc	pyruvate carboxylase	−13.1	1588	95
**Dgat2**	diacylglycerol O-acyltransferase 2	−12.4	4000	521
Cel	carboxyl esterase lipase	−12.1	955	79
Acacb	acetyl-Coenzyme A carboxylase beta	−11.5	530	46
**Acaa1b**	acetyl-Coenzyme A acyltransferase 1B	−10.6	696	66
**Ephx2**	epoxide hydrolase 2, cytoplasmic	−10.0	1402	140
**Lpl**	lipoprotein lipase	−9.6	6823	713
**Pgam2**	phosphoglycerate mutase 2	−8.9	628	70
**Cox6a2**	cytochrome c oxidase, subunit VI a, polypeptide 2	−8.1	405	50
**Fasn**	fatty acid synthase	−7.3	11558	1579
**Ptger3**	prostaglandin E receptor 3 (subtype EP3)	−7.1	1106	157
Sorbs1	sorbin and SH3 domain containing 1	−6.5	2532	581
Pygl	liver glycogen phosphorylase	−6.4	1600	250
**Scd1**	stearoyl-Coenzyme A desaturase 1	−6.4	7943	2026
Chpt1	choline phosphotransferase 1	−5.8	1658	327
**Slc1a5**	solute carrier family 1 (neutral amino acid transporter), member 5	−5.6	2664	476
**Acss2**	acyl-CoA synthetase short-chain family member 2	−5.5	969	160
**Mgll**	monoglyceride lipase	−5.5	3443	632
**Pnpla2**	patatin-like phospholipase domain containing 2	−5.1	4552	890
Eno3	enolase 3, beta muscle	−4.9	672	136
**Cyp2f2**	cytochrome P450 family 2, subfamily f, polypeptide 2	−4.9	550	112
**Lpin1**	lipin 1	−4.8	1167	268
Ido1	indoleamine 2,3-dioxygenase 1	−4.8	406	85
**Sod3**	superoxide dismutase 3, extracellular	−4.7	678	145
Cyp4b1	cytochrome P450 family 4, subfamily b, polypeptide 1	−4.6	1286	283
Igf1	insulin-like growth factor 1	−4.3	558	153
Aacs	acetoacetyl-CoA synthetase	−4.1	1176	320
**Acox1**	acyl-Coenzyme A oxidase 1, palmitoyl	−4.1	920	225
Xdh	xanthine dehydrogenase	−3.9	1400	362
**Gpd1**	glycerol-3-phosphate dehydrogenase 1 (soluble)	−3.6	1710	283
Gpt2	glutamic pyruvate transaminase (alanine aminotransferase) 2	−3.6	1338	438
Gpt	glutamic pyruvic transaminase, soluble	−3.6	577	159
Abca8a	ATP-binding cassette, sub-family A (ABC1), member 8a	−3.5	1675	478
**Me1**	malic enzyme 1, NADP(+)-dependent, cytosolic	−3.4	2810	900
Aqp1	aquaporin 1	−3.4	2841	848
Retsat	retinol saturase (all trans retinol 13,14 reductase)	−3.3	488	146
**Slc27a1**	solute carrier family 27 (fatty acid transporter), member 1	−3.2	522	163
**Lipa**	lysosomal acid lipase A	−3.2	374	117
Fads3	fatty acid desaturase 3	−3.2	1545	485
**Alox12e**	arachidonate lipoxygenase, epidermal	−3.1	818	262
**Elovl6**	ELOVL family member 6, elongation of long chain fatty acids (yeast)	−3.1	1088	320
Gpam	glycerol-3-phosphate acyltransferase, mitochondrial	−3.0	2818	947
Nr1h3	nuclear receptor subfamily 1, group H, member 3 (LXR)	−3.0	1137	379
Acly	ATP citrate lyase	−2.9	993	343
Pik3r1	phosphatidylinositol 3-kinase, regulatory subunit, polypeptide 1 (p85 alpha)	−2.9	558	192
Rbp7	retinol binding protein 7, cellular	−2.9	1212	418
**Slc2a4**	solute carrier family 2 (faciltated glucose transporter), member 4	−3.2	522	163
**Crat**	carnitine acetyltransferase	−2.8	537	191
**Slc2a4**	solute carrier family 2 (facilitated glucose transporter), member 4	−2.8	1021	364
Sord	sorbitol dehydrogenase	−2.8	700	250
Ehhadh	enoyl-Coenzyme A, hydratase/3-hydroxyacyl Coenzyme A dehydrogenase	−2.7	341	126
Hk2	hexokinase 2	−2.7	1447	534
Lpgat1	lysophosphatidylglycerol acyltransferase 1	−2.7	399	150
Gbe1	glucan (1,4-alpha-)branching enzyme	−2.7	723	267
Apod	apolipoprotein D	−2.6	4011	1526
Gatm	glycine amidinotransferase (L-arginine:glycine amidinotransferase	−2.6	400	152
Ltc4s	leukotriene C4 synthase	−2.6	467	179
Pfkfb1	6-phosphofructo-2-kinase/fructose-2,6-biphosphatase 1	−2.6	320	124
Plin2	perilipin 2	−2.5	6773	2671
**Cebpa**	CCAAT/enhancer binding protein (C/EBP), alpha	−2.5	1819	734
**Dgat1**	diacylglycerol O-acyltransferase 1	−2.5	1065	425
Ptgs1	prostaglandin-endoperoxide synthase 1	−2.5	362	145

**Table 2:** Gene ontology of differentially expressed genes affected by GW9662 Shown are enrichment data with P<0.05 by Fisher's Exact test.

Name	Total Entities	Overlap	Overlapping Entities	p-value
**DOWNREGULATED:**				
lipid metabolism	342	23	CHPT1,CD36,LEP,LPL,LIPE,APOD,NR1H3,SLC27A1,LIPA,ACSL1,HSD11B1,DGAT2,CRAT,ACLY,LPIN1,ACOX1,EHHADH,PNPLA2,PCK1,PNPLA3,MGLL,AACS,FADS3	1.92E-26
metabolism	858	21	LPGAT1,FASN,LIPE,ACACB,SLC27A1,EPHX2,ACSL1,GPAM,HSD11B1,ME1,PC,ACLY,PFKFB1,ACOX1,EHHADH,PNPLA2,GPD1,PNPLA3,ACSS2,PGAM2,AACS	3.71E-15
transport	1807	15	AQP1,CD36,APOD,SLC2A4,SLC27A1,FABP4,FABP3,CRAT,SORBS1,SLC1A5,AQP7,RBP4,CRABP1,RBP7,FADS3	6.01E-05
oxidation reduction	702	14	CYP2E1,PTGS1,SOD3,XDH,FASN,HSD11B1,ME1,ACOX1,EHHADH,GPD1,CYP4B1,SORD,RETSAT,FADS3	3.58E-09
fatty acid metabolism	110	12	CD36,PPARG,SLC27A1,LIPA,FABP4,ACSL1,GPAM,FABP3,CRAT,ACOX1,EHHADH,AACS	1.21E-16
response to drug	295	10	ADIPOQ,PPARG,LIPE,ACACB,FABP4,ACSL1,AQP7,SORD,ENO3,AACS	5.40E-09
response to insulin	37	10	LEP,RETN,PIK3R1,PFKFB1,PCK1,RBP4,PPARG,SORBS1,LPIN1,NRIH3	5.81E-10
fat cell differentiation	29	9	ADIG,CEBPA,ADIPOQ,PPARG,SLC2A4,FABP4,IGF1,AACS,RETN	1.21E-10
lipid biosynthesis	115	8	PTGS1,FASN,DGAT2,PC,ACLY,ELOVL6,ACSS2,FADS3	7.24E-10
gluconeogenesis	33	7	GPT,PC,PFKFB1,GPD1,PCK1,RBP4,PGAM2	2.60E-12
generation of precursor metabolites & energy	63	7	CEBPA,ADIPOQ,GYS2,ACOX1,AQP7,GBE1,COX6A2	3.17E-10
response to glucocorticoids	95	6	CEBPA,ADIPOQ,IGF1,FABP4,PIK3R1,PFKFB1	1.93E-07
response to nutrients	117	6	CEBPA,ADIPOQ,PPARG,ACSL1,GATM,AACS	6.62E-07
lipid catabolism	113	6	CEL,LPL,LIPE,LIPA,PNPLA2,PNPLA3	5.40E-07
glucose homeostasis	50	6	ADIPOQ,PPARG,SLC2A4,PCK1,RBP4,PYGL	3.87E-09
spermatogenesis	353	5	ADIG,ACOX1,AQP7,RBP4,PGAM2	2.38E-03
carbohydrate metabolism	296	5	SLC2A4,ME1,GPD1,PYGL,GBE1	1.10E-03
fatty acid biosynthesis	78	5	PTGS1,FASN,ACACB,ELOVL6,FADS3	1.98E-06
triglyceride biosynthesis	11	5	LPL,GPAM,DGAT1,DGAT2,PNPLA3	4.97E-11
glucose metabolism	115	5	ADIPOQ,LEP,HK2,PIK3R1,SORD	1.33E-05
inflammatory response	293	4	PPARG,LIPA,EPHX2,MGLL	7.45E-03
lung development	106	4	CEBPA,LIPA,HSD11B1,RBP4	1.78E-04
organ regeneration	49	4	CEBPA,PPARG,LPIN1,PFKFB1	8.49E-06
triglyceride catabolism	13	4	LPL,LIPE,PNPLA2,PNPLA3	3.08E-08
fatty acid beta-oxidation	32	4	ADIPOQ,FABP3,ACOX1,EHHADH	1.49E-06
glycolysis	68	4	HK2,PFKFB1,ENO3,PGAM2	3.14E-05
regulation of transcription	159	4	CEBPA,NR1H3,FABP4,PPARG	8.53E-03
response to ethanol	83	3	ADIPOQ,RBP4,AACS	1.37E-03
long-chain fatty acid transport	12	3	CD36,PPARG,FABP3	3.76E-06
aging	101	3	PTGS1,PIK3R1,ENO3	2.41E-03
fatty acid oxidation	18	3	CD36,ADIPOQ,PPARG	1.38E-05
glycogen metabolism	41	3	GYS2,PYGL,GBE1	1.72E-04
phospholipid biosynthesis	49	3	LPGAT1,CHPT1,GPAM	2.94E-04
regulation of cell proliferation	135	3	PTGS1,CEBPA,IGF1	5.44E-03
negative regulation of foam cell differentiation	10	3	ADIPOQ,PPARG,NR1H3	2.06E-06
**UPREGULATED:**				
regulation of transcription	2501	9	ZBTB16,MAPK8,RHOX5,BRWD1,ESRRB,RBM39,TARDBP,NFIB,THRAP3	2.24E-02
RNA splicing	238	5	HNRNPA1,PABPC1,RBM39,TARDBP,RBMX	2.13E-05
mRNA processing	277	5	PABPN1,HNRNPA1,PABPC1,RBM39,TARDBP	4.40E-05
cell proliferation	324	4	PTHLH,EREG,ZBTB16,NFIB	1.13E-03
central nervous system development	140	3	ZBTB16,PCP4,NPTX1	1.04E-03
translational elongation	161	3	RPS25,RPS24,RPL41	1.55E-03
cell-cell signaling	275	3	CALCA,PTHLH,EREG	6.96E-03
apoptosis	550	3	ZBTB16,SLC5A8,NISCH	4.29E-02

## DISCUSSION

The present study was designed to determine if pharmacological inhibition of PPARγ could sensitize mammary tumor growth to antiestrogen therapy. This concept was based on our previous finding that induction of mammary carcinogenesis in transgenic mice expressing the dominant-negative Pax8PPARγ fusion protein resulted in increased ER expression and responsiveness to the ER antagonist, fulvestrant [10]. MMTV-Pax8PPARγ transgenic mice represent a rare mouse model in which the mammary gland exhibits a progenitor cell phenotype that results in the preferential development of ER^+^ rather than ER^−^ tumors of mixed lineage following progestin/DMBA treatment [10, 28]. A similar mammary tumor phenotype developed in caveolin-1 knockout mice that was also associated with the induction of several stem/progenitor cell markers, including RBM39 [27], as found in the present study. RBM39 functions primarily in RNA splicing and may also be a putative partner of the co-activator Ncoa6/PRIP [29]. Thus, one unexpected finding was that GW9662 upregulated a number of genes associated with transcription, processing, splicing and translation that likely contribute to the diversity of the proteome [30].

GW9662 is an irreversible PPARγ antagonist [16], although *in vitro* cell studies have also reported off-target effects [24-26]. However, there are no *in vivo* studies that have established whether GW9662 is PPARγ-selective. In one instance, GW9662 was shown to reduce high fat diet-induced obesity in rats when administered in the diet at a concentration of 0.1% [31], which was identical to the GW9662 diet used in our study. GW9662 was also shown to block the anti-inflammatory effects of the PPARγ agonist, rosiglitazone, in endotoxin-induced acute lung injury after intravenous administration [32]. Based on gene array profiling, we found that GW9662 elicited PPARγ specificity based on its direct and indirect inhibitory effects on the expression of metabolic genes known to be under the control of PPARs.

An important caveat to the use of GW9662 is its ability to induce a modest acceleration of tumorigenesis when administered orally at the onset of carcinogenesis. We also observed a similar effect in MMTV-Pax8PPARγ mice following progestin/DMBA mammary carcinogenesis [10]. While this has not been reported previously, the ability of GW9662 to *inhibit* cell growth *in vitro* similarly to PPARγ agonists [24, 33, 34] suggests the presence of “off-target” effects. The increase in tumorigenesis observed with GW9662 and the dominant-negative Pax8PPARγ transgene suggests that partial antagonists rather than full antagonists or drugs with greater specificity may be a useful approach for further studies. Clearly, additional pharmacokinetic and pharmacodynamic studies *in vivo* are needed to establish the bioavailability and metabolic effects of GW9662. Overall, the positive aspect of inhibiting PPARγ was its ability to sensitize tumors to the ER antagonist fulvestrant, suggesting the potential for such an approach for hormone-insensitive malignancies.

## MATERIALS AND METHODS

### Animal model

FVB wild-type (WT) mice were obtained from Taconic Farms, Germantown, N.Y. All animal studies were conducted under protocols approved by the Georgetown University Animal Care and Use Committee.

### Mammary carcinogenesis

Five week-old WT mice were treated with medroxyprogesterone acetate and DMBA as previously described [9, 28]. Briefly, mice were injected s.c. with 15 mg medroxyprogesterone acetate suspension (Depo-Provera?), and after seven days were administered four weekly doses of 1 mg DMBA/0.1 ml cottonseed oil by gavage. One day after the last dose of DMBA, mice were divided into four groups of 10 mice each: 1) one group was maintained on standard Purina Rodent Chow 5001, 2) one group was maintained on chow supplemented with 0.1% (w/w) GW9962, 2) one group was maintained on chow supplemented with GW9662 and injected s.c. every other week with 250 mg/kg fulvestrant (Faslodex^®^) and 4) one group was injected with 250 mg/kg fulvestrant every other week. GW9662 was provided by the Chemoprevention Branch, NCI. The histopathology of the resulting tumors is presented in Table S1.

### Antibodies

The source of antibodies, their dilution and use were the following: rabbit anti-ERα (sc-542, Santa Cruz Biotechnology, 1:200 for IHC, 1:1,000 for western); rabbit anti-PgR (sc-538, Santa Cruz Biotechnology, 1:200 for IHC, 1:1,000 for western).

### Immunohistochemistry

IHC analysis was carried out as previously described [9, 10, 28].

### Western Blotting

Western blotting was carried out as previously described [10]. Briefly, tissue was frozen in liquid nitrogen and pulverized in a mortar and pestle, and mixed with lysis buffer containing: 0.1% SDS, 0.5% NP-40, phenylmethylsulfonyl fluoride, 1 mM sodium vanadate, 50 mM sodium fluoride, 10 mM β-glycerophosphate, 5 mM sodium pyrophosphate, and protease inhibitor cocktail (Roche Diagnostics). Following incubation on ice for 30 min, lysates were cleared by centrifugation for 15 min at 13,000 x g at 4°C. Protein concentrations were determined by the Coomassie Plus Protein Assay (Pierce), and 50 μg of lysate was separated in a 4-12% NuPAGE Bis-Tris gel (Invitrogen). After wet transfer, membranes were blocked for 1 hr at room temperature in TBS (pH 7.4) containing 5% non-fat dry milk and 0.1% Tween 20. Primary antibody was incubated overnight at 4°C, and secondary antibody was incubated for 1 hr at room temperature. Proteins were visualized with either SuperSignal West Pico or SuperSignal West Dura (Pierce).

### Gene Microarray Analysis

Total RNA was extracted using an RNeasy Mini Kit (Qiagen) following the manufacturer's protocol as previously described [10, 35]. cRNA was synthesized using the Affymetrix (Santa Clara, CA) protocol with minor modifications as described [28]. Biotin-labeled cRNA was fragmented for 35 min at 94°C and hybridized overnight to an Affymetrix mouse 430A 2.0 GeneChip^®^ representing approximately 22,000 annotated mouse genes by the Genomics and Epigenomics Shared Resource, Lombardi Comprehensive Cancer Center, Georgetown University. Hybridization signals were detected with an Agilent Gene Array scanner, and grid alignment and raw data generation performed with Affymetrix GeneChip^®^ Operating software 1.1. Changes in gene expression with a signal ≥300 (log2 ≥8.1) and ≥3-fold change [9, 35, 36] were clustered hierarchically with CIMiner software (National Cancer Institute, NIH). Array data are presented in Table S2, and complete data files were deposited in the GEO database under accession no. GSE33762.

### Quantitative Real-Time Polymerase Chain Reaction (qRT-PCR)

Total RNA was extracted using the RNAeasy Mini Kit (Qiagen, Valencia, CA) according to the manufacturer's protocol as previously described [10, 35]. One μg of RNA was reverse transcribed in a total volume of 20 μl using the Cloned AMV First-Strand cDNA Synthesis kit (Invitrogen). PCR was performed in triplicate in an ABI 7900 instrument (Applied Biosystems, Foster City, CA) using SYBRGreen detection (Applied Biosystems, Foster City, CA) according to the manufacturer's protocol. qRT-PCR primers were designed using the primer design tool at http://www.idtdna.com/Scitools/Applications/RealTimePCR/. Efficiencies of all primer sets (Table S1) were validated using a standard curve of five serial cDNA dilutions in water in duplicate. Primers were acceptable if the deviation from the slope of the standard curve was <0.3, and if the melting curve showed only one product. The expression of each target gene was normalized to the expression of GAPDH, and the relative quantification method was applied using SDS2.3 software (Applied Biosystems, Foster City, CA). Primers are listed in Table S3.

### Statistical Analysis

Survival curves were analyzed by Pearson's log rank test and cumulative tumor formation by Student's two-tailed t test at a significance level of *P*≤0.05.

## SUPPLEMENTARY TABLES


