# Erratum: Drug-Targeted Inhibition of Peroxisome Proliferator-Activated Receptor-gamma Enhances the Chemopreventive Effect of Anti-Estrogen Therapy

**DOI:** 10.18632/oncotarget.26161

**Published:** 2018-09-18

**Authors:** Hongyan Yuan, Levy Kopelovich, Yuzhi Yin, Jin Lu, Robert I. Glazer

**Affiliations:** ^1^ Department of Oncology, Georgetown University School of Medicine, and Lombardi Comprehensive Cancer Center, Washington, DC; ^2^ Chemoprevention Branch, National Cancer Institute, Bethesda, MD; ^3^ Laboratory of Allergic Diseases, National Institute of Allergy and Infectious Diseases, Bethesda, MD

**This article has been corrected:** During production, the ending page number for this article was listed incorrectly. The page count has now been adjusted to show the proper pagination.

Original article: Oncotarget. 2012; 3:345-356.https://doi.org/10.18632/oncotarget.457

