# Supplementary material for: Drug-Targeted Inhibition of Peroxisome Proliferator-Activated Receptorγ Enhances the Chemopreventive Effect of Anti-Estrogen
Source: Oncotarget. 2012 Apr 13;3(3):345–56. doi: 10.18632/oncotarget.457 (PMC3359890; doi:10.18632/oncotarget.457)
Supplement: Supplementary file 1 [file oncotarget-03-345-s001.pdf]

SUPPLEMENTARY DATA

Yuan et al. 2012. Drug-Targeted Inhibition of Peroxisome Proliferator-Activated Receptor-gamma Enhances the Chemopreventive Effect of Anti-Estrogen Therapy

**Table S1. Tumor histopathology.** Mice were fed standard rodent chow or chow supplemented with 0.1% GW9662 beginning one day after the last dose of DMBA. The number of tumors and the percentage of each histotype is indicated. The was a significant difference between GW9662-treated animals vs. fulvestrant (*P* =0.0333), but not between GW9662-treated animals vs.control. (*P* =0.0867). There was no significant difference between control vs. fulvestrant. Statistical analysis was by Fisher's Exact test. There was an insufficient number of tumor-bearing animals treated with GW9662 and fulvestrant for statistical analysis.

| Tumor Histology | Control | GW9662 | Fulvestrant | GW9662+Fulv |
|---|---|---|---|---|
| Adenocarcinoma | 5 (50%) | 9 (90%) | 4 (40%) | 1 (100%) |
| Adenosquamous/Squamous | 1 (10%) | 1 (10%) | 2 (20%) | 0 |
| Myoepithelial | 4 (40%) | 0 | 4 (40%) | 0 |

**Table S2. Gene expression in adenocarcinomas from control and GW9662-treated wild-type mice.**
Animals were maintained on a diet containing 0.1% GW9662 beginning one day after the last dose of DMBA as described in Fig. 1. Shown are genes whose raw values were ≥300 and changed ≥2.5-fold in GW9662-treated animals vs. control mice.

| | | | Raw Value | |
|---|---|---|---|---|
| **Gene symbol** | **Gene Title** | **Fold Change** | **Control** | **GW9662** |
| Ces3 | carboxylesterase 3 | -105.7 | 2733 | 25 |
| Tmem45b | transmembrane protein 45b | -89.3 | 3775 | 42 |
| Gys2 | glycogen synthase 2 | -74.7 | 558 | 7 |
| Lep | leptin | -74.7 | 1231 | 16 |
| Adrb3 | adrenergic receptor, beta 3 | -69.3 | 644 | 9 |
| Csn1s2a | casein alpha s2-like A | -69.0 | 8326 | 583 |
| Cidec | cell death-inducing DFFA-like effector c | -60.6 | 2171 | 36 |
| Aqp7 | aquaporin 7 | -55.6 | 3523 | 63 |
| Mrap | melanocortin 2 receptor accessory protein | -55.5 | 699 | 13 |
| Pnpla3 | patatin-like phospholipase domain containing 3 | -53.9 | 1301 | 24 |
| Cox8b | cytochrome c oxidase, subunit VIIIb | -51.0 | 1324 | 26 |
| Amy1 | amylase 1, salivary | -45.1 | 1721 | 38 |
| Cyp2e1 | cytochrome P450, family 2, subfamily e, polypeptide 1 | -44.3 | 5209 | 118 |
| Slc7a10 | solute carrier family 7 (cationic amino acid transporter, y+ system), member 1C | -43.9 | 560 | 13 |
| Pck1 | phosphoenolpyruvate carboxykinase 1, cytosolic | -43.9 | 3071 | 70 |
| Wap | whey acidic protein | -43.7 | 7058 | 161 |
| Sycp3 | synaptonemal complex protein 3 | -43.7 | 1954 | 45 |
| Retn | resistin | -35.7 | 10637 | 298 |
| Orm1 | orosomucoid 1 | -34.5 | 1083 | 31 |
| Rbp4 | retinol binding protein 4, plasma | -33.9 | 3187 | 94 |
| Plin4 | perilipin 4 | -33.4 | 3181 | 95 |
| Mc2r | melanocortin 2 receptor | -32.2 | 575 | 18 |
| Lao1 | L-amino acid oxidase 1 | -30.2 | 3092 | 103 |
| Adhfe1 | alcohol dehydrogenase, iron containing, 1 | -29.4 | 797 | 33 |
| Sncg | synuclein, gamma | -28.7 | 4718 | 164 |
| Fabp3 | fatty acid binding protein 3, muscle and heart | -27.0 | 886 | 33 |
| Thrsp | thyroid hormone responsive SPOT14 homolog (Rattus) | -24.8 | 7743 | 340 |
| Timp4 | tissue inhibitor of metalloproteinase 4 | -24.0 | 646 | 15 |
| Myoz1 | myozenin 1 | -23.8 | 5834 | 245 |
| Cd36 | CD36 antigen | -22.5 | 6726 | 306 |
| Cidea | cell death-inducing DNA fragmentation factor, alpha subunit-like effector A | -22.5 | 918 | 42 |
| Car4 | carbonic anhydrase 4 | -22.3 | 982 | 44 |
| Car3 | carbonic anhydrase 3 | -21.9 | 12543 | 1726 |
| Fabp4 | fatty acid binding protein 4, adipocyte | -21.7 | 7777 | 3543 |
| Myh1 | myosin, heavy polypeptide 1, skeletal muscle, adult | -21.7 | 857 | 40 |
| Adipoq | adiponectin, C1Q and collagen domain containing | -21.6 | 10299 | 522 |
| Adig | adipogenin | -20.8 | 1676 | 85 |
| Dmbt1 | deleted in malignant brain tumors 1 | -20.2 | 1155 | 57 |
| Lhx8 | LIM homeobox protein 8 | -20.2 | 555 | 26 |
| Acsl1 | acyl-CoA synthetase long-chain family member 1 | -18.6 | 3172 | 374 |
| Glycam1 | glycosylation dependent cell adhesion molecule 1 | -18.6 | 2953 | 166 |
| Npr3 | natriuretic peptide receptor 3 | -17.8 | 1362 | 77 |
| Ckmt2 | creatine kinase, mitochondrial 2 | -17.6 | 820 | 46 |
| Lalba | lactalbumin, alpha | -17.0 | 361 | 21 |
| Csn1s1 | casein alpha s1 | -16.2 | 14199 | 874 |
| Lipe | lipase, hormone sensitive | -16.2 | 1329 | 82 |
| Hsd11b1 | hydroxysteroid 11-beta dehydrogenase 1 | -15.7 | 2815 | 179 |
| Pcolce2 | procollagen C-endopeptidase enhancer 2 | -15.5 | 510 | 33 |
| Smr2 | submaxillary gland androgen regulated protein 2 | -15.2 | 523 | 43 |
| Csn2 | casein beta | -15.1 | 11308 | 1378 |
| Gdf10 | growth differentiation factor 10 | -15.1 | 400 | 26 |
| Klb | klotho beta | -15.1 | 629 | 42 |
| Ifi27l2b | interferon, alpha-inducible protein 27 like 2B | -14.8 | 683 | 46 |
| Ppp1r1a | protein phosphatase 1, regulatory (inhibitor) subunit 1A | -14.0 | 704 | 50 |
| Pparg | peroxisome proliferator activated receptor gamma | -13.9 | 967 | 69 |
| Atp2a1 | ATPase, Ca++ transporting, cardiac muscle, fast twitch 1 | -13.7 | 489 | 36 |
| Pcx | pyruvate carboxylase | -13.1 | 1588 | 95 |
| Gjb6 | gap junction protein, beta 6 | -12.6 | 565 | 45 |

| | | | | |
|---|---|---|---|---|
| Ckm | creatine kinase, muscle | -12.5 | 1380 | 110 |
| Inmt | indolethylamine N-methyltransferase | -12.5 | 3067 | 245 |
| Pvalb | parvalbumin | -12.4 | 636 | 51 |
| Dgat2 | diacylglycerol O-acyltransferase 2 | -12.4 | 4000 | 521 |
| Acta1 | actin, alpha 1, skeletal muscle | -12.1 | 3899 | 322 |
| Aldh1a7 | aldehyde dehydrogenase family 1, subfamily A7 | -12.1 | 1109 | 92 |
| Cel | carboxyl ester lipase | -12.1 | 955 | 79 |
| Hoxa5 | homeobox A5 | -11.9 | 336 | 28 |
| Aspa | aspartoacylase | -11.6 | 340 | 29 |
| Acacb | acetyl-Coenzyme A carboxylase beta | -11.5 | 530 | 46 |
| Nnat | neuronatin | -11.2 | 2663 | 237 |
| Tnnt3 | troponin T3, skeletal, fast | -11.2 | 778 | 69 |
| Tnnc2 | troponin C2, fast | -11.2 | 2204 | 197 |
| Myh4 | myosin, heavy polypeptide 4, skeletal muscle | -11.2 | 1428 | 128 |
| Fgb | fibrinogen beta chain | -11.0 | 3413 | 311 |
| Ifi27l2a | interferon, alpha-inducible protein 27 like 2A | -10.8 | 8124 | 750 |
| Acaa1b | acetyl-Coenzyme A acyltransferase 1B | -10.6 | 696 | 66 |
| Myl1 | myosin, light polypeptide 1 | -10.4 | 1656 | 166 |
| Aoc3 | amine oxidase, copper containing 3 | -10.3 | 3162 | 306 |
| Btn1a1 | butyrophilin, subfamily 1, member A1 | -10.3 | 1440 | 140 |
| Ephx2 | epoxide hydrolase 2, cytoplasmic | -10.0 | 1402 | 140 |
| Atp1a2 | ATPase, Na+/K+ transporting, alpha 2 polypeptide | -10.0 | 441 | 25 |
| Ntrk2 | neurotrophic tyrosine kinase, receptor, type 2 | -9.7 | 709 | 73 |
| Lpl | lipoprotein lipase | -9.6 | 6823 | 713 |
| Cfd | complement factor D (adipsin) | -9.5 | 11467 | 1203 |
| Pgam2 | phosphoglycerate mutase 2 | -8.9 | 628 | 70 |
| Nmu | neuromedin U | -8.3 | 1226 | 148 |
| Ebf3 | early B-cell factor 3 | -8.2 | 638 | 78 |
| Cox6a2 | cytochrome c oxidase, subunit VI a, polypeptide 2 | -8.1 | 405 | 50 |
| Fhl1 | four and a half LIM domains 1 | -8.0 | 1435 | 179 |
| Rarres2 | retinoic acid receptor responder (tazarotene induced) 2 | -8.0 | 3001 | 384 |
| Lgals12 | lectin, galactose binding, soluble 12 | -8.0 | 871 | 109 |
| Art3 | ADP-ribosyltransferase 3 | -8.0 | 1692 | 219 |
| Ctsr | cathepsin R | -7.9 | 3420 | 431 |
| Ltf | lactotransferrin | -7.9 | 2207 | 279 |
| Cpa3 | carboxypeptidase A3, mast cell | -7.7 | 603 | 79 |
| Fasn | fatty acid synthase | -7.3 | 11558 | 1579 |
| Hoxa3 | homeobox A3 | -7.3 | 379 | 52 |
| Igh-2 | immunoglobulin heavy chain 2 (serum IgA) | -7.3 | 2201 | 308 |
| Mylpf | myosin light chain, phosphorylatable, fast skeletal muscle | -7.3 | 1620 | 223 |
| G0s2 | G0/G1 switch gene 2 | -7.2 | 4310 | 598 |
| Ccl21a | chemokine (C-C motif) ligand 21A | -7.1 | 307 | 43 |
| Ptger3 | prostaglandin E receptor 3 (subtype EP3) | -7.1 | 1106 | 157 |
| Ghr | growth hormone receptor | -6.9 | 3255 | 695 |
| Hnmt | histamine N-methyltransferase | -6.9 | 409 | 59 |
| Pi16 | peptidase inhibitor 16 | -6.8 | 823 | 121 |
| Igj | immunoglobulin joining chain | -6.6 | 1353 | 204 |
| Fmo1 | flavin containing monooxygenase 1 | -6.5 | 876 | 135 |
| Slc22a3 | solute carrier family 22 (organic cation transporter), member 3 | -6.5 | 566 | 87 |
| Sorbs1 | sorbin and SH3 domain containing 1 | -6.5 | 2532 | 581 |
| Tns1 | tensin 1 | -6.5 | 637 | 97 |
| Enpep | glutamyl aminopeptidase | -6.4 | 1253 | 195 |
| Pygl | liver glycogen phosphorylase | -6.4 | 1600 | 250 |
| Scd1 | stearoyl-Coenzyme A desaturase 1 | -6.4 | 7943 | 2026 |
| Lims2 | LIM and senescent cell antigen like domains 2 | -6.2 | 408 | 65 |
| Mup1 | major urinary protein 1 | -6.2 | 709 | 114 |
| Aldh1l1 | aldehyde dehydrogenase 1 family, member L1 | -6.0 | 553 | 93 |
| Tnni2 | troponin I, skeletal, fast 2 | -5.9 | 1042 | 228 |
| Chpt1 | choline phosphotransferase 1 | -5.8 | 1658 | 327 |
| Lrg1 | leucine-rich alpha-2-glycoprotein 1 | -5.8 | 1110 | 191 |
| Ccdc80 | coiled-coil domain containing 80 | -5.7 | 2600 | 944 |
| Eepd1 | endonuclease/exonuclease/phosphatase family domain containing 1 | -5.6 | 323 | 57 |
| Fzd4 | frizzled homolog 4 (Drosophila) | -5.6 | 413 | 74 |
| Slc1a5 | solute carrier family 1 (neutral amino acid transporter), member 5 | -5.6 | 2664 | 476 |
| Acss2 | acyl-CoA synthetase short-chain family member 2 | -5.5 | 969 | 160 |

| | | | | |
|---|---|---|---|---|
| Prelp | proline arginine-rich end leucine-rich repeat | -5.5 | 1378 | 249 |
| Mgll | monoglyceride lipase | -5.5 | 3443 | 632 |
| Nrg4 | neuregulin 4 | -5.4 | 683 | 126 |
| Dpt | dermatopontin | -5.3 | 3830 | 724 |
| Mettl7a1 | methyltransferase like 7A1 | -5.2 | 1873 | 352 |
| Sult1a1 | sulfotransferase family 1A, phenol-preferring, member 1 | -5.2 | 1688 | 323 |
| Chst1 | carbohydrate (keratan sulfate Gal-6) sulfotransferase 1 | -5.1 | 1227 | 240 |
| Igfals | insulin-like growth factor binding protein, acid labile subunit | -5.1 | 306 | 59 |
| Nnmt | nicotinamide N-methyltransferase | -5.1 | 872 | 171 |
| Pnpla2 | patatin-like phospholipase domain containing 2 | -5.1 | 4552 | 890 |
| Clec3b | C-type lectin domain family 3, member b | -5.0 | 1419 | 284 |
| Scg3 | secretogranin III | -5.0 | 428 | 85 |
| Calml3 | calmodulin-like 3 | -4.9 | 458 | 93 |
| Cyp2f2 | cytochrome P450, family 2, subfamily f, polypeptide 2 | -4.9 | 550 | 112 |
| Eno3 | enolase 3, beta muscle | -4.9 | 672 | 136 |
| Ido1 | indoleamine 2,3-dioxygenase 1 | -4.8 | 406 | 85 |
| Ldb3 | LIM domain binding 3 | -4.8 | 426 | 89 |
| Lpin1 | lipin 1 | -4.8 | 1167 | 268 |
| Gstz1 | glutathione transferase zeta 1 (maleylacetoacetate isomerase) | -4.7 | 1602 | 342 |
| Sod3 | superoxide dismutase 3, extracellular | -4.7 | 678 | 145 |
| Cyp4b1 | cytochrome P450, family 4, subfamily b, polypeptide 1 | -4.6 | 1286 | 283 |
| Fxyd1 | FXYD domain-containing ion transport regulator 1 | -4.6 | 734 | 158 |
| Higd1c | HIG1 domain family, member 1C | -4.6 | 1027 | 232 |
| Slc24a3 | solute carrier family 24 (sodium/potassium/calcium exchanger), member 3 | -4.6 | 821 | 179 |
| Smoc1 | SPARC related modular calcium binding 1 | -4.5 | 697 | 154 |
| Fam20c | family with sequence similarity 20, member C | -4.5 | 803 | 179 |
| Mmd | monocyte to macrophage differentiation-associated | -4.5 | 4007 | 898 |
| Htra3 | HtrA serine peptidase 3 | -4.4 | 1619 | 367 |
| Adh1 | alcohol dehydrogenase 1 (class I) | -4.3 | 835 | 194 |
| Igf1 | insulin-like growth factor 1 | -4.3 | 558 | 153 |
| Mbp | myelin basic protein | -4.3 | 1266 | 186 |
| Steap4 | STEAP family member 4 | -4.3 | 2348 | 546 |
| Tmprss2 | transmembrane protease, serine 2 | -4.3 | 1582 | 514 |
| Krt79 | keratin 79 | -4.2 | 530 | 127 |
| Mfng | MFNG O-fucosylpeptide 3-beta-N-acetylglucosaminyltransferase | -4.2 | 451 | 108 |
| Odz4 | odd Oz/ten-m homolog 4 (Drosophila) | -4.2 | 336 | 81 |
| Ogn | osteoglycin | -4.2 | 960 | 339 |
| Sgce | sarcoglycan, epsilon | -4.2 | 516 | 122 |
| Aacs | acetoacetyl-CoA synthetase | -4.1 | 1176 | 320 |
| Acox1 | acyl-Coenzyme A oxidase 1, palmitoyl | -4.1 | 920 | 225 |
| Cmbl | carboxymethylenebutenolidase-like (Pseudomonas) | -4.1 | 1116 | 275 |
| Gjb2 | gap junction protein, beta 2 | -4.1 | 1572 | 384 |
| Cldn15 | claudin 15 | -4.0 | 513 | 129 |
| Ndrg1 | N-myc downstream regulated gene 1 | -4.0 | 518 | 144 |
| Ppp1r3c | protein phosphatase 1, regulatory (inhibitor) subunit 3C | -4.0 | 864 | 214 |
| Pxmp2 | peroxisomal membrane protein 2 | -4.0 | 597 | 148 |
| Tcap | titin-cap | -4.0 | 324 | 80 |
| Tmem120b | transmembrane protein 120B | -4.0 | 492 | 123 |
| Depdc6 | DEP domain containing 6 | -3.9 | 589 | 149 |
| Pdlim3 | PDZ and LIM domain 3 | -3.9 | 1138 | 294 |
| Prnp | prion protein | -3.9 | 1258 | 319 |
| Smoc2 | SPARC related modular calcium binding 2 | -3.9 | 1025 | 263 |
| Xdh | xanthine dehydrogenase | -3.9 | 1400 | 362 |
| Abi3bp | ABI gene family, member 3 (NESH) binding protein | -3.8 | 351 | 92 |
| Dbp | D site albumin promoter binding protein | -3.8 | 1275 | 371 |
| Ifi203 | interferon activated gene 203 | -3.8 | 434 | 114 |
| Aldh6a1 | aldehyde dehydrogenase family 6, subfamily A1 | -3.7 | 2191 | 585 |
| C3 | complement component 3 | -3.7 | 4416 | 1208 |
| Hp | haptoglobin | -3.7 | 4266 | 1141 |
| Palmd | palmdelphin | -3.7 | 681 | 182 |
| Rbpms | RNA binding protein gene with multiple splicing | -3.7 | 467 | 125 |
| Smr3a | submaxillary gland androgen regulated protein 3A | -3.7 | 980 | 264 |
| Adamts5 | a disintegrin-like and metallopeptidase (reprolysin type) with thrombospondin type 1 motif, 5 | -3.6 | 649 | 179 |
| Ebf1 | early B-cell factor 1 | -3.6 | 780 | 219 |
| Gpd1 | glycerol-3-phosphate dehydrogenase 1 (soluble) | -3.6 | 1710 | 283 |

| | | | | |
|---|---|---|---|---|
| Gpt | glutamic pyruvic transaminase, soluble | -3.6 | 577 | 159 |
| Gpt2 | glutamic pyruvate transaminase (alanine aminotransferase) 2 | -3.6 | 1338 | 438 |
| Klf15 | Kruppel-like factor 15 | -3.6 | 532 | 149 |
| Txnip | thioredoxin interacting protein | -3.6 | 2044 | 570 |
| Abca8a | ATP-binding cassette, sub-family A (ABC1), member 8a | -3.5 | 1675 | 478 |
| Fgf13 | fibroblast growth factor 13 | -3.5 | 471 | 136 |
| Gnai1 | guanine nucleotide binding protein (G protein), alpha inhibiting 1 | -3.5 | 2034 | 738 |
| Ifi205 | interferon activated gene 205 | -3.5 | 1089 | 311 |
| Net1 | neuroepithelial cell transforming gene 1 | -3.5 | 647 | 185 |
| Sfrp2 | secreted frizzled-related protein 2 | -3.5 | 618 | 174 |
| Aqp1 | aquaporin 1 | -3.4 | 2841 | 848 |
| Cxcr7 | chemokine (C-X-C motif) receptor 7 | -3.4 | 1152 | 337 |
| Dpep1 | dipeptidase 1 (renal) | -3.4 | 768 | 229 |
| Gstt1 | glutathione S-transferase, theta 1 | -3.4 | 1040 | 308 |
| Ly6d | lymphocyte antigen 6 complex, locus D | -3.4 | 4415 | 1301 |
| Me1 | malic enzyme 1, NADP(+)-dependent, cytosolic | -3.4 | 2810 | 900 |
| Gata2 | GATA binding protein 2 | -3.3 | 492 | 151 |
| Igfbp6 | insulin-like growth factor binding protein 6 | -3.3 | 1466 | 450 |
| Mb | myoglobin | -3.3 | 747 | 230 |
| Prkch | protein kinase C, eta | -3.3 | 332 | 101 |
| Rbpms2 | RNA binding protein with multiple splicing 2 | -3.3 | 791 | 237 |
| Retsat | retinol saturase (all trans retinol 13,14 reductase) | -3.3 | 488 | 146 |
| Spp1 | secreted phosphoprotein 1 | -3.3 | 5518 | 1673 |
| Thbd | thrombomodulin | -3.3 | 559 | 170 |
| Timp3 | tissue inhibitor of metalloproteinase 3 | -3.3 | 1895 | 613 |
| Bglap | bone gamma carboxyglutamate protein | -3.2 | 489 | 154 |
| Cd1d1 | CD1d1 antigen | -3.2 | 1837 | 638 |
| Dram1 | DNA-damage regulated autophagy modulator 1 | -3.2 | 1239 | 393 |
| Efemp1 | epidermal growth factor-containing fibulin-like extracellular matrix protein 1 | -3.2 | 1758 | 543 |
| Fads3 | fatty acid desaturase 3 | -3.2 | 1545 | 485 |
| Fndc1 | fibronectin type III domain containing 1 | -3.2 | 681 | 214 |
| Hoxc8 | homeobox C8 | -3.2 | 386 | 120 |
| Lipa | lysosomal acid lipase A | -3.2 | 374 | 117 |
| Nid1 | nidogen 1 | -3.2 | 543 | 172 |
| Slc25a10 | solute carrier family 25 (mitochondrial carrier, dicarboxylate transporter), member 10 | -3.2 | 336 | 104 |
| Slc27a1 | solute carrier family 27 (fatty acid transporter), member 1 | -3.2 | 522 | 163 |
| Alox12e | arachidonate lipoxygenase, epidermal | -3.1 | 818 | 262 |
| Cldn5 | claudin 5 | -3.1 | 1127 | 362 |
| Elovl6 | ELOVL family member 6, elongation of long chain fatty acids (yeast) | -3.1 | 1088 | 320 |
| Fap | fibroblast activation protein | -3.1 | 857 | 280 |
| Spnb2 | spectrin beta 2 | -3.1 | 440 | 142 |
| St3gal6 | ST3 beta-galactoside alpha-2,3-sialyltransferase 6 | -3.1 | 302 | 99 |
| Ang3 | angiogenin, ribonuclease A family, member 3 | -3.0 | 2978 | 1004 |
| Gpam | glycerol-3-phosphate acyltransferase, mitochondrial | -3.0 | 2818 | 947 |
| Hoxd8 | homeobox D8 | -3.0 | 487 | 162 |
| Ndrg2 | N-myc downstream regulated gene 2 | -3.0 | 944 | 314 |
| Nr1h3 | nuclear receptor subfamily 1, group H, member 3 | -3.0 | 1137 | 379 |
| Selenbp1 | selenium binding protein 1 | -3.0 | 1699 | 559 |
| Slc1a3 | solute carrier family 1 (glial high affinity glutamate transporter), member 3 | -3.0 | 638 | 175 |
| Acly | ATP citrate lyase | -2.9 | 993 | 343 |
| Aplp2 | amyloid beta (A4) precursor-like protein 2 | -2.9 | 1405 | 481 |
| Cdkn1c | cyclin-dependent kinase inhibitor 1C (P57) | -2.9 | 756 | 263 |
| Galnt2 | UDP-N-acetyl-alpha-D-galactosamine:polypeptide N-acetylgalactosaminyltransferase 2 | -2.9 | 345 | 118 |
| Pik3r1 | phosphatidylinositol 3-kinase, regulatory subunit, polypeptide 1 (p85 alpha) | -2.9 | 558 | 192 |
| Ptprb | protein tyrosine phosphatase, receptor type, B | -2.9 | 369 | 125 |
| Rbp7 | retinol binding protein 7, cellular | -2.9 | 1212 | 418 |
| Rasgrp2 | RAS, guanyl releasing protein 2 | -2.8 | 502 | 178 |
| Ak3 | adenylate kinase 3 | -2.8 | 681 | 243 |
| C1ra | complement component 1, r subcomponent A | -2.8 | 1063 | 384 |
| Crat | carnitine acetyltransferase | -2.8 | 537 | 191 |
| Fst | follistatin | -2.8 | 688 | 246 |
| Hspb8 | heat shock protein 8 | -2.8 | 564 | 203 |
| Ms4a4d | membrane-spanning 4-domains, subfamily A, member 4D | -2.8 | 390 | 142 |
| Pam | peptidylglycine alpha-amidating monooxygenase | -2.8 | 1019 | 363 |
| Papss2 | 3'-phosphoadenosine 5'-phosphosulfate synthase 2 | -2.8 | 445 | 161 |

| | | | | |
|---|---|---|---|---|
| Slc2a4 | solute carrier family 2 (facilitated glucose transporter), member 4 | -2.8 | 1021 | 364 |
| Sord | sorbitol dehydrogenase | -2.8 | 700 | 250 |
| Akr1c14 | aldo-keto reductase family 1, member C14 | -2.7 | 433 | 158 |
| Atrx | alpha thalassemia/mental retardation syndrome X-linked homolog (human) | -2.7 | 389 | 146 |
| Car2 | carbonic anhydrase 2 | -2.7 | 1269 | 464 |
| Col13a1 | collagen, type XIII, alpha 1 | -2.7 | 465 | 172 |
| Ehd2 | EH-domain containing 2 | -2.7 | 1388 | 507 |
| Ehhadh | enoyl-Coenzyme A, hydratase/3-hydroxyacyl Coenzyme A dehydrogenase | -2.7 | 341 | 126 |
| Entpd2 | ectonucleoside triphosphate diphosphohydrolase 2 | -2.7 | 559 | 205 |
| Gbe1 | glucan (1,4-alpha-), branching enzyme 1 | -2.7 | 723 | 267 |
| Hk2 | hexokinase 2 | -2.7 | 1447 | 534 |
| Kcnk5 | potassium channel, subfamily K, member 5 | -2.7 | 398 | 147 |
| Lbp | lipopolysaccharide binding protein | -2.7 | 1028 | 377 |
| Lpgat1 | lysophosphatidylglycerol acyltransferase 1 | -2.7 | 399 | 150 |
| Nos3 | nitric oxide synthase 3, endothelial cell | -2.7 | 419 | 158 |
| Pecam1 | platelet/endothelial cell adhesion molecule 1 | -2.7 | 582 | 213 |
| Pmp22 | peripheral myelin protein 22 | -2.7 | 3041 | 1133 |
| Serping1 | serine (or cysteine) peptidase inhibitor, clade G, member 1 | -2.7 | 3517 | 1320 |
| Alas2 | aminolevulinic acid synthase 2, erythroid | -2.6 | 471 | 180 |
| Apod | apolipoprotein D | -2.6 | 4011 | 1526 |
| Atp6v1c2 | ATPase, H+ transporting, lysosomal V1 subunit C2 | -2.6 | 1170 | 451 |
| C4b | complement component 4B (Childo blood group) | -2.6 | 1144 | 445 |
| Clca2 | chloride channel calcium activated 2 | -2.6 | 1831 | 713 |
| Gatm | glycine amidinotransferase (L-arginine:glycine amidinotransferase) | -2.6 | 400 | 152 |
| Ltc4s | leukotriene C4 synthase | -2.6 | 467 | 179 |
| Mxra8 | matrix-remodelling associated 8 | -2.6 | 1386 | 535 |
| Pfkfb1 | 6-phosphofructo-2-kinase/fructose-2,6-biphosphatase 1 | -2.6 | 320 | 124 |
| Slc12a2 | solute carrier family 12, member 2 | -2.6 | 1279 | 490 |
| Slc39a8 | solute carrier family 39 (metal ion transporter), member 8 | -2.6 | 578 | 222 |
| Snta1 | syntrophin, acidic 1 | -2.6 | 370 | 141 |
| Car6 | carbonic anhydrase 6 | -2.6 | 400 | 156 |
| Ckb | creatine kinase, brain | -2.6 | 1135 | 445 |
| Alpl | alkaline phosphatase, liver/bone/kidney | -2.5 | 388 | 154 |
| Aspn | asporin | -2.5 | 357 | 142 |
| Cebpa | CCAAT/enhancer binding protein (C/EBP), alpha | -2.5 | 1819 | 734 |
| Clca1 | chloride channel calcium activated 1 | -2.5 | 1773 | 698 |
| Dcn | decorin | -2.5 | 9261 | 3670 |
| Dgat1 | diacylglycerol O-acyltransferase 1 | -2.5 | 1065 | 425 |
| Folr1 | folate receptor 1 (adult) | -2.5 | 711 | 285 |
| Gstm1 | glutathione S-transferase, mu 1 | -2.5 | 1918 | 753 |
| H19 | H19 fetal liver mRNA | -2.5 | 566 | 223 |
| Idh1 | isocitrate dehydrogenase 1 (NADP+), soluble | -2.5 | 1532 | 616 |
| Mknk2 | MAP kinase-interacting serine/threonine kinase 2 | -2.5 | 437 | 176 |
| Opn3 | opsin 3 | -2.5 | 395 | 160 |
| Plin2 | perilipin 2 | -2.5 | 6773 | 2671 |
| Ptgs1 | prostaglandin-endoperoxide synthase 1 | -2.5 | 362 | 145 |
| Ptrf | polymerase I and transcript release factor | -2.5 | 2562 | 1027 |
| Serpina3n | serine (or cysteine) peptidase inhibitor, clade A, member 3N | -2.5 | 797 | 324 |
| Vegfb | vascular endothelial growth factor B | -2.5 | 438 | 176 |
| Gsn | gelsolin | -2.5 | 9034 | 3380 |
| Slc6a15 | solute carrier family 6 (neurotransmitter transporter), member 15 | 2.5 | 289 | 709 |
| Arl4d | ADP-ribosylation factor-like 4D | 2.5 | 323 | 797 |
| Tcfap2b | transcription factor AP-2 beta | 2.5 | 248 | 614 |
| Serinc1 | serine incorporator 1 | 2.5 | 125 | 315 |
| Ereg | epiregulin | 2.5 | 125 | 315 |
| Rps25 | ribosomal protein S25 | 2.5 | 459 | 1159 |
| Brwd1 | bromodomain and WD repeat domain containing 1 | 2.5 | 165 | 417 |
| Aard | alanine and arginine rich domain containing protein | 2.6 | 187 | 480 |
| Luc7l2 | LUC7-like 2 (S. cerevisiae) | 2.6 | 143 | 453 |
| Meg3 | maternally expressed 3 | 2.6 | 277 | 722 |
| Mapk8 | mitogen-activated protein kinase 8 | 2.6 | 187 | 490 |
| Pabpn1 | poly(A) binding protein, nuclear 1 | 2.6 | 480 | 1266 |
| Cwc22 | CWC22 spliceosome-associated protein homolog (S. cerevisiae) | 2.7 | 460 | 1244 |
| Eppk1 | epiplakin 1 | 2.7 | 567 | 1530 |
| Rbmx | RNA binding motif protein, X chromosome | 2.7 | 220 | 596 |

| | | | | |
|---|---|---|---|---|
| Rpl41 | ribosomal protein L41 | 2.7 | 1250 | 3419 |
| Zbtb16 | zinc finger and BTB domain containing 16 | 2.8 | 550 | 1536 |
| Esrrb | estrogen related receptor, beta | 2.8 | 179 | 505 |
| Snhg1 | small nucleolar RNA host gene (non-protein coding) 1 | 2.9 | 132 | 380 |
| Vps41 | vacuolar protein sorting 41 (yeast) | 2.9 | 111 | 320 |
| Zfp207 | zinc finger protein 207 | 2.9 | 168 | 490 |
| Tardbp | TAR DNA binding protein | 3.0 | 257 | 759 |
| Nisch | nischarin | 3.0 | 151 | 487 |
| Rps24 | ribosomal protein S24 | 3.0 | 184 | 559 |
| Tesc | tescalcin | 3.1 | 122 | 372 |
| Ints6 | integrator complex subunit 6 | 3.1 | 485 | 1490 |
| Pabpc1 | poly(A) binding protein, cytoplasmic 1 | 3.1 | 130 | 406 |
| Bex1 | brain expressed gene 1 | 3.2 | 1306 | 4129 |
| Gas5 | growth arrest specific 5 | 3.2 | 276 | 888 |
| Cdc7 | cell division cycle 7 (S. cerevisiae) | 3.2 | 300 | 971 |
| Pthlh | parathyroid hormone-like peptide | 3.5 | 161 | 556 |
| Rhox5 | reproductive homeobox 5 | 3.5 | 156 | 546 |
| Thrap3 | thyroid hormone receptor associated protein 3 | 3.5 | 130 | 458 |
| Nptx1 | neuronal pentraxin 1 | 3.6 | 325 | 1183 |
| Crabp1 | cellular retinoic acid binding protein I | 3.8 | 858 | 3278 |
| Calca | calcitonin/calcitonin-related polypeptide, alpha | 4.0 | 437 | 1747 |
| Wdr26 | WD repeat domain 26 | 4.2 | 108 | 452 |
| Il33 | interleukin 33 | 4.2 | 1112 | 4699 |
| Slc5a8 | solute carrier family 5 (iodide transporter), member 8 | 4.3 | 358 | 1553 |
| Lpp | LIM domain containing preferred translocation partner in lipoma | 4.4 | 88 | 385 |
| Nfib | nuclear factor I/B | 4.4 | 148 | 649 |
| Tug1 | Taurine upregulated gene 1 | 4.4 | 120 | 527 |
| Bex2 | brain expressed X-linked 2 | 4.4 | 114 | 503 |
| Psmd7 | proteasome (prosome, macropain) 26S subunit, non-ATPase, 7 | 4.5 | 77 | 349 |
| Pcp4 | Purkinje cell protein 4 | 4.5 | 1503 | 6826 |
| Wfdc12 | WAP four-disulfide core domain 12 | 4.7 | 636 | 2961 |
| Bex4 | brain expressed gene 4 | 4.8 | 260 | 1241 |
| Phip | pleckstrin homology domain interacting protein | 4.8 | 65 | 312 |
| Hnrnpa1 | heterogeneous nuclear ribonucleoprotein A1 | 5.4 | 187 | 1013 |
| Rbm39 | RNA binding motif protein 39 | 6.6 | 96 | 596 |
| Mt1 | metallothionein 1 | 6.8 | 300 | 2043 |
| D2Ertd93e | DNA segment, Chr 2, ERATO Doi 93, expressed | 7.0 | 131 | 919 |
| Rasgrf1 | RAS protein-specific guanine nucleotide-releasing factor 1 | 11.1 | 146 | 1253 |

**Table S3. Primers used for qRT-PCR analysis**

| Gene | Forward Primer (5'→3') | Reverse Primer (5'→3') |
|---|---|---|
| PPARγ | CAGACAAGATTTGAAAGAAG | CAGAATGGCATCTCTGTGTC |
| Esr1 | AACCGCCCATGATCTATTCTG | AGATTCAAGTCCCCAAAGCC |
| Pgr | TGAGCCTGATGGTGTTTGG | ACAGCGAGTAGAATGACAGC |
| Fabp4 | GCCTTTCTCACCTGGAAGAC | CCCACTCCCACTTCTTTCAT |
| Fasn | GGGTTCTAGCCAGCAGAGTC | TCAGCCACTTGAGTGTCCTC |
| Lep | GACATTTCACACACGCAGTC | GTGAAGCCCAGGAATGAAGT |
